# Intelligence, Correspondence, &c.

**Published:** 1835-10-01

**Authors:** 


### INTELLIGENCE, CORRESPONDENCE, &c.

### NEW THEATRE OF ANATOMY, KINNERTON STREET,

*Adjoining St. George's Hospital.*

This splendid Anatomical School will be opened on the 1st October, with an Introductory Lecture, by Mr. Tatum. For convenience of arrangement, and spacious accommodations, this extensive building is unrivalled. It is contiguous to the Hospital, within the walls of which all Lectures (save those on Anatomy) are delivered by the Medical Officers of the Institution.

### PORTRAIT OF DR. JOHNSON.

Recently published by Mr. Highley, 32, Fleet Street, price 10s. 6d. (five shillings to Subscribers) a highly finished Engraving by Phillips, from a painting by J. Wood, of Dr. James Johnson, Editor of the Medico-Chirurgical Review.

" An elegantly executed Mezzotinto, the *vera effigies* of our cotemporary."—*Med. Gazette.*

☞ The painting and engraving alone cost upwards of one hundred guineas. Any Subscriber to the Medico-Chirurgical Review, will be entitled to a proof engraving for the mere expense of the paper and press-work, by sending the following form to Mr. Highley.

*Mr. Highley is requested to deliver to the bearer of this a proof-print of the Portrait of Dr. James Johnson, price five shillings.*

1*st October*, 1835.

This form is printed in the advertising sheet so as to be cut out.

### GENERAL INDEX TO THE MEDICO-CHIRURGICAL REVIEW.

This highly useful volume, price 3s. 6d., has been printed at a great expense, and Subscribers are requested to order copies early, as only a thousand copies are struck off, and they are getting low.

*His Majesty's Ship* Tyne, *Gibraltar, 6th June,* 1835.

Sir,—The Case of Mr. Sprague, advertized in the last Number of your Review, having appeared to me, to call loudly for pecuniary support from the benevolent of all classes, but especially from his professional brethren, I have much pleasure in contributing my mite towards his relief. It is also with much gratification that I likewise add, that after submitting the case to the consideration of my messmates, they very cheerfully subscribed as follows:

| | |
|---|---|
| Mr. Wellesley, one dollar. | A young Gentleman, two dollars. |
| Mr. Murray, one dollar. | Mr. Compton, one dollar. |
| A young Gentleman, one dollar. | Mr. Kingston, two dollars. |
| Mr. Wilcox, five shillings. | Mr. Law, five shillings. |
| Mr. Jinkins, one dollar. | Mr. Coote, one dollar. |
| Mr. King, five shillings. | Mr. Malme, one dollar. |
| Mr. Clendon, one dollar. | Mr. Parrish, one dollar. |
| Mr. M'Naghton, one dollar. | Mr. Symms, one dollar. |

Mr. Henry, one dollar, in addition to my guinea previously transmitted.

Total amount of subscriptions from the Midshipmen's Berth of His Majesty's Ship Tyne, *Five Guineas.*

I am, Sir, Yours truly, J. Plimsole,

*Assistant Surgeon, H. M. S. Tyne.*

